# Semi-supervised action recognition using logit aligned consistency and adaptive negative learning

**DOI:** 10.1038/s41598-025-01922-2

**Published:** 2025-05-30

**Authors:** Fengyun Zuo, Yang Xu, Minggang Wang

**Affiliations:** 1 College of Big Data and Information Engineering, Guizhou University, Guiyang, 550025 China; 2 Zunyi Aluminum Stock Corporation Ltd, Zunyi, 563100 China

**Keywords:** Adaptive negative learning, Logit standardization preprocessing, Negative labels, Semi-supervised action recognition, Vision transformer, Computer science, Information technology

## Abstract

With the development of the socialized video era, while semi-supervised action recognition can address the increasingly high costs of video annotation, it still faces significant challenges, particularly in the underexplored application of Vision Transformer. In this paper, we present our work on designing Full-SVFormer, a simple yet efficient semi-supervised action recognition architecture based on the Transformer framework. Full-SVFormer uses TimeSformer based on pre-trained weights as its backbone network, which balances the accuracy and speed of Transformers in semi-supervised action recognition. Within the stable pseudo-label framework EMA-Teacher, we introduce KL divergence loss, which has undergone logit standardization preprocessing, as its unsupervised consistency loss. This improves the student’s focus on the inherent relationship between the logit of student and teacher. Furthermore, we incorporate the Adaptive Negative Learning (ANL) method to introduce additional negative pseudo-labels, which dynamically evaluate the Top-k performance of the model to adaptively assign negative labels, thus making better use of ambiguous prediction examples. We conducted a number of experiments on two extensive datasets, UCF-101 and HMDB-51, where our overall experimental results achieved superior performance compared to previous methods. Our work further advances the development of Transformer in the domain of semi-supervised action recognition.

## Introduction

Human actions serve as expressions of thoughts and emotions in everyday life, and recognizing such actions has found widespread applications in health monitoring, intelligent surveillance, sports analysis and exercise, and autonomous driving^1–3^. In recent years, with the continuous maturation of technologies like 5G, AI, AR, and VR, as well as their expansive applications, video content is poised to become a cornerstone of the internet’s social era. The volume of video content is expected to grow exponentially, with platforms like YouTube and TikTok witnessing tens of millions of new uploads daily. This surge poses a significant challenge in obtaining annotated videos at a large scale. Thus, leveraging large volumes of unlabeled videos for better video understanding holds great research significance^4–6^.

Semi-supervised action recognition^4,7,8^ has garnered immense attention for its ability to mitigate reliance on large labeled datasets, enabling efficient utilization of vast unlabeled data. One common approach is to assign pseudo-labels to unlabeled data. Assigning pseudo-labels to unlabeled data is a typical method used to predict the unlabeled data after the model training. The predictions with high confidence are then utilized as pseudo-labels for the respective videos, which helps improve the network’s training. The generated pseudo-labels’ quality significantly influences the effectiveness of this method. To enhance pseudo-label quality and supervise unlabeled samples, additional modalities have been incorporated into existing methods, such as temporal gradients^8^, optical flow^9,10^, and auxiliary networks^11–13^. Although the above methods achieved satisfactory results, the additional training or enormous inference costs they needed hindered their scalability for in-depth research.

In the current research, video Transformer^14–16^ have outperformed CNNs^17–19^ in the domain of visual action recognition. However, studies^20^ indicate that Transformers, due to their lack of inductive bias common in vision tasks, typically require extensive training data and longer training times, resulting in subpar performance under low-data conditions compared to CNNs^21^. Applying semi-supervised learning methods like FixMatch^22^ directly to Vision Transformers (ViT)^23^ usually leads to performance degradation. To resolve this problem, Zhen Xing et al.^24^ observed that initializing TimeSformer^15^ with ImageNet^25^ weights yielded promising results even with limited labeled data. This observation led to the innovative SVFormer, which combines the popular video Transformer architecture TimeSformer, a stable pseudo-label framework EMA-Teacher^26^, and novel data augmentation strategies, which provide a strong solution for semi-supervised action recognition.

However, this framework typically uses the cross-entropy loss as the unsupervised consistency loss function within the EMA-Teacher distillation framework. The cross-entropy loss is directly designed for classification tasks, providing gradients that improve learning and reflect class probabilities. In the context of distillation learning, the Kullback–Leibler (KL) divergence loss is utilized to gauge the distinction between two probability distributions. This approach effectively conveys rich information about inter-class and intra-class relationships from the teacher model’s soft la-bels. Additionally, in distillation methods based on logit-based KL divergence loss^27,28^, a temperature parameter is often introduced to adjust the teacher model’s outputs, producing smoother probability distributions that help the student model learn more generalized feature representations. Consequently, using KL divergence loss as the unsupervised loss function in a knowledge distillation algorithm can serve a regularization role, preventing the student model from overfitting to noise. Sun et al.^29^ point out that cross-entropy loss and KL divergence loss are equivalent when optimizing the student’s predictions independently. Traditional knowledge distillation methods assume that a temperature parameter is shared between the teacher and student models, which implies that their logits’ range and variance must match perfectly. Given the capacity differences between the student and teacher models, this enforced matching would limit the student model’s performance. Based on these aforementioned issues, we propose that when employing a KL divergence-based distillation method in semi-supervised action recognition, it is crucial to standardize the logit prediction distributions of both the teacher and student models. This standardization approach can accelerate the training process of the student model, enhance its generalization capability, and lead to significant performance improvements.

In addition, research by Kim et al.^30^ pointed out that when a model is trained using noisy data, it may lead to training errors, poor classification performance, and thus severely degraded performance. Therefore, they pro-posed a framework that combines negative learning, which can filter out noisy data for correct training. FullMatch^31^ pointed out that the pseudo-labels of some semi-supervised learning methods, such as FixMatch, must be filtered out by a high threshold to filter out noisy pseudo-labels. This practice does not fully utilize unlabeled sample data, resulting in a waste of complex examples. In semi-supervised action recognition, to better leverage all unlabeled samples, we introduced a new method of Adaptive Negative Learning (ANL), which introduces additional negative pseudo-labels for all unlabeled data. The adjustment of this label is carried out by dynamically assessing the model’s Top-k performance to make use of examples with low confidence. As a semi-supervised learning meth-od, EMA-Teacher’s generation of pseudo-labels also re-quires filtration using a high threshold, which may not contribute during the training stage of the model. By combining with ANL, it can present negative pseudo-labels of all unlabeled data for training with very limited additional computational overhead, consequently enhancing the model’s predictive accuracy.

**Fig. 1:**
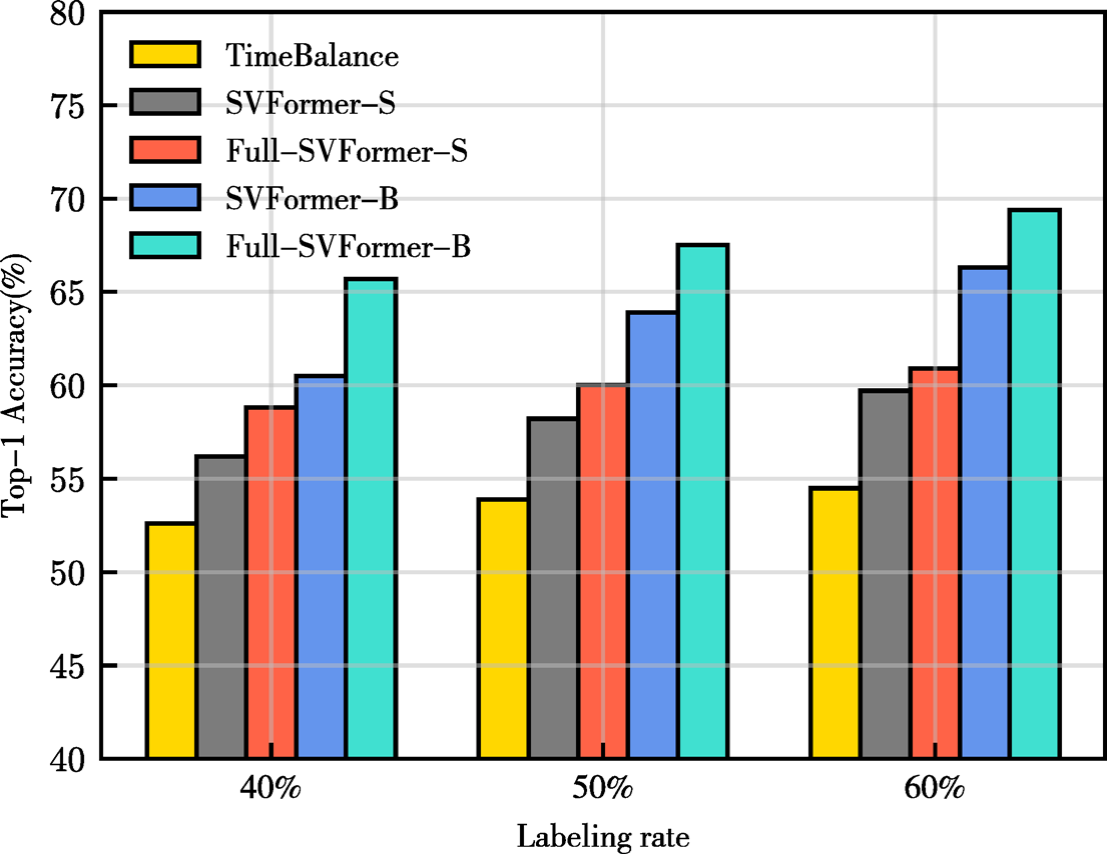
The Top-1 accuracy performance of different semi-supervised action recognition models at various labeling rates on the HMDB-51 dataset.

To improve the performance of the Transformer architecture in semi-supervised action recognition, this paper presents a framework called Full-SVFormer, shown in Fig. 1, which notably enhances the model’s performance. In summary, the main contributions of this work are as follows:

1. We have innovatively introduced logit standardization preprocessing to enhance the unsupervised loss of KL divergence of the EMA-Teacher. Standardizing the prediction distributions of both teacher and student models can significantly improve model performance in the domain of semi-supervised action recognition.
2. Within the sphere of semi-supervised action recognition, we have integrated Adaptive Negative Learning (ANL) with the semi-supervised recognition framework EMA-Teacher for the first time. This method effectively allocates and utilizes negative pseudo-labels for all unlabeled data, including low-confidence data, and demonstrates strong performance in optimizing the model.
3. We have developed a straightforward and efficient framework called Full-SVFormer. This framework has been tested extensively on two video action recognition benchmark datasets. This framework generally outperforms state-of-the-art methods in overall performance for both Full-SVFormer-B and Full-SVFormer-S models of different sizes.

## Related work

### Deep semi-supervised learning

Deep learning models’ construction and training heavily rely on large-scale annotated datasets. Nevertheless, the process of collecting and annotating such datasets is often complex and time-consuming. With the increase in the cost of annotation and the explosion of data volumes, semi-supervised learning (SSL) has been widely applied to model training using small numbers of labeled samples and large numbers of unlabeled samples that are readily available. Computer vision is one of the primary application domains for SSL. For example, Das et al^32^. proposed AnoMed, a semi-supervised framework that integrates the novel Scale-Invariant Bottleneck (SIB) and a confidence guided pseudo-label optimizer (PLO). In particular, data augmentation and consistency regularization, which are central to SSL, reduce the necessity for labeled data and enhance generalization ability and performance of model. Specifically, different augmented views^33^ of the data are inputted into the model, and consistency loss is used to enforce the alignment of outputs from these different augmented views. Additionally, various data mixing augmentations^34–36^ enrich the variety of sample forms, allowing the generated pseudo-labels to provide extra training signals, while consistency loss ensures the reliability of these extra signals. Among these advanced methods, FixMatch^22^ is notable for its effectiveness, with its variants being applied to numerous other tasks. Compared to the traditional FixMatch method, EMA-Teacher^26^ offers more stable training behavior and better results. Generally, the cross-entropy loss is pivotal in the consistency regularization of EMA-Teacher, and it is theoretically equivalent to logit-based KL divergence loss^29^. The KL divergence, as a method for measuring the difference between two probability distributions, makes it particularly suitable for evaluating the similarity between the probability distributions of model predictions and actual data. In our work, KL divergence is used as the loss function in consistency regularization, which aligns with the EMA-Teacher algorithm’s framework.

### Semi-supervised action recognition

Past methods have explored action recognition using both 2D^7^ and 3D^8,12,37,38^ convolutional networks. For instance, Videossl^4^ proposed the combination of CNNs with pseudo-labels and the probability predictions of image classifiers as guidance signals for unlabeled data in the video domain, effectively enhancing video classification performance. ActorCutMix^39^ introduced an innovative scene enhancement strategy that significantly improved the data efficiency of semi-supervised learning in the domain of action recognition, achieving excellent effects. MvPL^38^ leverages the optical flow modality from two complementary perspectives appearance and motion to obtain more reliable pseudo-labels. This method also encourages the model to learn distinctive regions^40^ by aligning local actors with their corresponding scene information. LTG^8^ introduces the time gradient modality for generating high-quality pseudo-labels for training. CMPL^12^ incorporates a lightweight auxiliary network that works alongside the main network to jointly predict pseudo-labels in video clips. ActNetFormer^13^ combines an auxiliary network with contrastive learning to enable models to gather stronger spatial–temporal representations. TimeBalance^41^ combines two teachers using a time similarity reweighting scheme to utilize unlabeled videos, with temporal invariance and specificity. Although these methods have achieved commendable overall performance, they typically require more video frames or training cycles, making the training process challenging. SVFormer^24^ is the initial video Transformer used for semi-supervised action recognition, which obtained the highest performance with the least amount of training required. For semi-supervised action recognition, this model set a new standard and has stimulated additional research in this field using the Transformer architecture. Therefore, we have made enhancements built upon this groundwork and put forward Full-SVFormer.

### Video transformer

Vision Transformers (ViT)^23^ have demonstrated their potential in computer vision tasks, achieving significant successes in areas such as image generation^42^, semantic segmentation^43^, and object detection^44^. This success has spurred deep exploration into video understanding tasks based on the Transformer architecture. ViViT^16^ is the first video-understanding model using a pure Trans-former architecture. By exploring relationships between spatiotemporal tokens, shows promise in handling video data effectively. VidTr^45^ introduces separable attention for video classification, stacking attention mechanisms to aggregate spatiotemporal information, thus providing better performance with higher efficiency. VTN^46^ ditches the traditional reliance on 2D convolutional networks for video action recognition, instead focusing on information from the entire video sequence to improve the model’s capability to handle lengthy video sequences. Timesformer^15^ implements various spatiotemporal attention mechanisms, balancing speed and accuracy via spatiotemporal separable attention. Additionally, research such as MviT^47^, Video Swin Transformer^14^, Uniformer^48^, and Video Mobile-Former^49^ introduce inductive biases into Transformers, primarily focusing on fully supervised set-tings. In contrast, SVFormer^24^, based on video Trans-former, through innovative data augmentation techniques like TubeTokenMix and a stable pseudo-label framework named EMA-Teacher, offers a powerful solution for semi-supervised action recognition. This work encourages further research in this area.

### Knowledge distillation

Knowledge distillation’s key concept^50^ is that the classifier (commonly referred to as a student) is trained based on the output from another classifier (commonly referred to as a teacher), using these outputs as soft labels. This approach accelerates the student model’s learning process and enhances its performance. Throughout the student’s training period, its prediction probability is aligned with the softmax output of the logit by minimizing the KL divergence loss between the probability distributions of the model predictions and those of the teacher model. As one of the three main categories of response-based knowledge distillation^51^, this approach employs “temperature parameters” to adjust the degree of softening of the teacher model’s outputs. This adjustment enables the student model to achieve a smoother probability distribution, thereby enhancing its generalization capability. Several studies^50,52,53^ have explored this concept, concluding that the level of temperature affects the degree of focus on negative labels during the training process of student. Lower temperatures typically result in less attention to negative labels, particularly those significantly low-er than average, while higher temperatures increase the importance of negative labels, prompting the student to pay more attention to them. By narrowing the discrepancy in the predicted probability distributions between the teacher and student, ATKD^54^ introduces a sharpness index to select an adaptive temperature. However, these studies have not thoroughly explored the source of temperature or the potential for different temperature allocations. Sun et al.^29^ analytical inference based on the principle of entropy maximization demonstrates that students and teachers do not necessarily share temperature. They also introduced the temperature to the weighted standard deviation of logit and perform Z-score normalization preprocessing before applying softmax and KL divergence. This method alleviates the problem where traditional logit-based KD distillation algorithms implicitly require precise matching between the logits of teacher and student, promoting improvements in existing logit-based KD methods. Therefore, this paper introduces logit normalization preprocessing of the prediction distribution in the existing EMA-Teacher framework. This approach can enhance the distillation effect and accelerate the convergence of model training.

### Negative learning

Negative learning^31^ is an indirect training method where models are trained using categories of input images that do not belong to the supplementary labels of supervised learning. Unlike "positive labels," negative labels have the advantage of being cheaper and more accurate. Current methods still often rely on “high confidence” predictions even from low probability values. For example, techniques such as UPS^55^ and NS^3^ L^56^ choose negative labels for categories with probability values lower than a specific small threshold. However, this approach fails to label samples with negative labels when predictions are ambiguous. Adaptive Negative Learning (ANL)^31^ addresses this limitation by focusing on category ranking rather than probability values for negative label allocation. ANL dynamically allocates negative labels to unlabeled samples without introducing additional threshold hyperparameters, thereby maintaining the simplicity of model training. In our work, adaptive negative learning is integrated into the mixed training framework TTMix of SVFormer^24^. This approach allows for the effective use of negative samples throughout the entire training process, enhancing the model’s generalization and discrimination abilities.

## Methodology

**Fig. 2:**
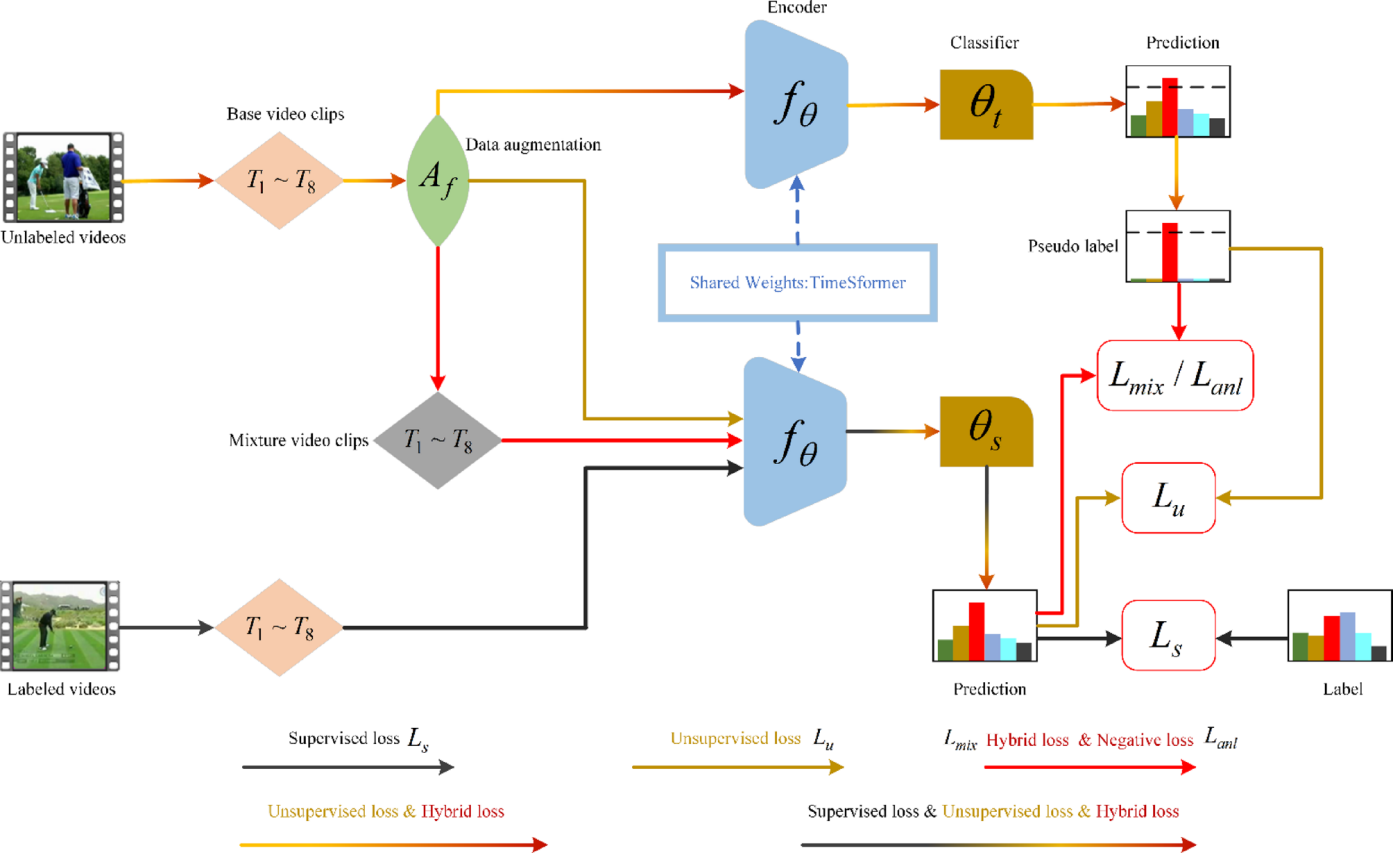
The overall architecture of Full-SVFormer, which encompasses both supervised loss training with labeled data and unsupervised or semi-supervised hybrid loss training with unlabeled data.

We will begin by introducing the fundamental principles of SSL in section A. Following that, we outline the training framework utilized in section B. In section C, we introduce a novel method for performing logit normalization on the EMA-Teacher’s teacher and student prediction distributions. section D discusses an effective yet straightforward mixed-sample adaptive negative learning (ANL). Finally, we illustrate the training paradigm examined in this study, as depicted in Fig. 2, in section E.

### SSL preliminaries

Assume the dataset contains N training video samples, which includes ${N}_{L}$ labeled video samples $({x}_{l},{y}_{l})\in {D}_{L}$ and ${N}_{u}$ unlabeled video samples ${x}_{u}\in {D}_{U}$. Here, ${x}_{l}$ represents the labeled video samples and their corresponding labels ${y}_{l}$, while ${x}_{u}$ represents unlabeled video samples. Additionally, there is a relationship expressed as ${N}_{U}>>{N}_{L}$ between ${N}_{L}$ and ${N}_{U}$. The SSL preparatory video dataset ${N}_{L}$ and ${N}_{U}$ must satisfy these conditions in order to effectively train the model.

### Semi-supervised action recognition framework

#### Full-SVFormer supervised loss

**Fig. 3:**
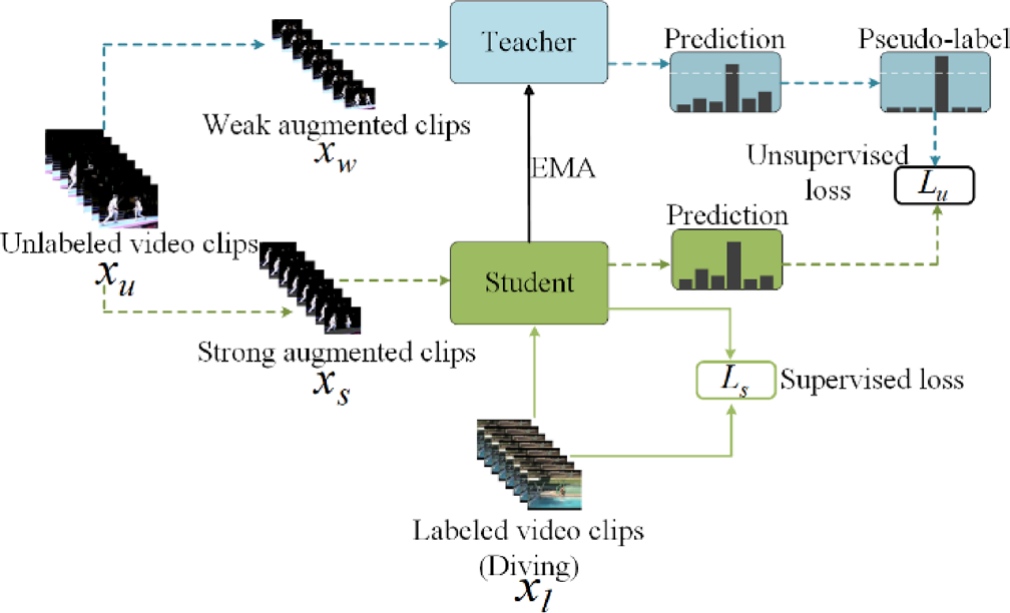
The semi-supervised training framework of Full-SVFormer.

The semi-supervised training framework presented in this paper employs an enhanced version of EMA-Teacher, built upon FixMatch. This framework integrates the pseudo-label algorithm with consistency regularization between two different data augmentation views. The training paradigm incorporates both supervised loss ${\mathcal{L}}_{s}$ and unsupervised loss ${\mathcal{L}}_{u}$. In terms of labeled dataset, the supervised loss ${\mathcal{L}}_{s}$ is optimized by the model as shown in Fig. 3:

1 $$\begin{array}{c}{\mathcal{L}}_{s}=\frac{1}{{N}_{L}}\sum^{{N}_{L}}\mathcal{H}\left(\mathcal{F}\left({x}_{l}\right),{y}_{l}\right)\end{array}$$

where $\mathcal{F}(\mathcal{*})$ denotes the predicted class generated by the model for the labeled video data, and $\mathcal{H}(\mathcal{*})$ represents the standard cross-entropy loss.

#### Full-SVFormer unsupervised loss

For the unlabeled sample ${x}_{u}$, the unsupervised loss training framework is shown in Fig. 3. First, two views are generated using weak and strong data augmentation techniques. The view ${x}_{w}={\mathcal{A}}_{\text{weak }}({x}_{u})$ is obtained by applying weak augmentation techniques such as random scaling, random cropping, and random horizontal flip to ${x}_{u}$. The view ${x}_{s}={\mathcal{A}}_{\text{strong}}({x}_{u})$ is generated using strong augmentation techniques such as AutoAugment^33^ or Dropout^57^. Then, the pseudo-label ${\widehat{y}}_{w}=arg max({\mathcal{F}}_{t}({x}_{w}))$ generated by the teacher model ${\mathcal{F}}_{t}$ on the weak view ${x}_{w}$ is used to train the student model ${\mathcal{F}}_{s}$ on the strong view ${x}_{s}$. The unsupervised consistency loss ${\mathcal{L}}_{u}$ is defined as follows:

2 $$\begin{array}{c}{\mathcal{L}}_{u}=\frac{1}{{N}_{U}}\sum^{{N}_{U}}{\mathbb{I}}\left(max\left({\mathcal{F}}_{t}\left({x}_{w}\right)\right)>\delta \right)\mathcal{H}\left({\mathcal{F}}_{s}\left({x}_{s}\right),{\widehat{y}}_{w}\right)\end{array}$$

where $\delta$ is a threshold of predefined confidence, and ${\mathbb{I}}$ is an indicator that equals 1 when the maximum class prediction probability of the model on ${x}_{w}$ exceeds $\delta$, otherwise it equals 0. The confidence indicator effectively filters out noisy pseudo-labels for training. There is an enhanced version of FixMatch named the exponential moving average teacher (EMA-Teacher) model, adopted by the ${\mathcal{F}}_{t}$. This approach addresses the issue of model collapse in FixMatch caused by the same model being shared by two augmented inputs^58^ It not only generates stable pseudo-labels, but it also updates the model parameters by using the exponential moving average of the student model parameters, expressed as follows:

3 $$\begin{array}{c}{\theta }_{t}\leftarrow m{\theta }_{t}+\left(1-m\right){\theta }_{s}\end{array}$$

where $m$ is the momentum coefficient hyperparameter, and ${\theta }_{t}$ and ${\theta }_{s}$ are the parameters of the model of teacher and student, respectively. There is significant success that EMA has achieved not only in object detection, self-supervised learning, and image classification in SSL but also in the semi-supervised action recognition tasks of SVFormer^24^.

#### Full-SVFormer mixed supervised loss

**Fig. 4:**
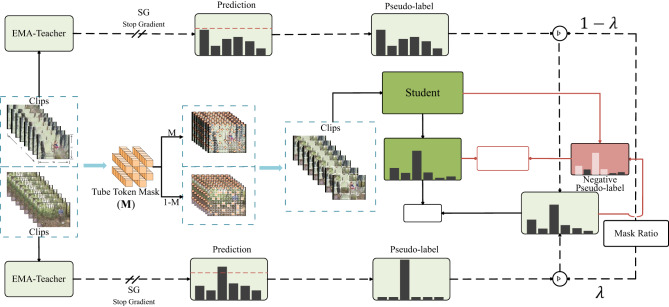
Overview of hybrid training framework and adaptive negative learning training strategy. Just to note, “SG” stands for the stop gradient, showing where the gradient flow is stopped in the process. By using a tube mask, the TTMix training framework takes two input unlabeled samples and mixes them. The resulting mixed sample is then used to train the student model. In parallel, the EMA-Teacher model gets the original samples to generate pseudo-labels for each sample. For the mixed sample, these pseudo-labels are subsequently linearly interpolated to generate a pseudo-label using the mask ratio λ. Otherwise, we use negative pseudo-labels (burgundy bars) for all unlabeled mixed samples using the proposed ANL approach. Data augmentation steps are not shown here for simplicity.

One of the core challenges in SSL is generating high-quality pseudo-labels to enhance the dataset. Mixup^59^, a widely adopted data augmentation strategy, along with its variant Cutmix^60^, has proven effective in generating pseudo-labels by mixing unlabeled samples in image classification. However, the SVFormer framework has shown that, like Mixup and Cutmix, pixel-level mixing augmentation strategies may not be well-suited for token-level models when using Transformers as the backbone network. To address this limitation, SVFormer proposed token-level mixing augmentation methods specifically for video data: Tube TokenMix, Rand TokenMix, and Frame TokenMix. These methods generally outperform pixel-level mixing methods, with Tube TokenMix (hereafter referred to as TTMix) significantly leading in overall performance. Given TTMix’s outstanding results, this study continues to utilize it for generating high-quality pseudo-labels in SSL. As illustrated in Fig. 4, TTMix is based on a token-level mask-mixing strategy. For a given unlabeled video clip ${x}_{a},{x}_{b}\in {\mathbb{R}}^{H\times W\times T}$, where T is the total number of frames, and H and W represent the height and width of the frames following patch tokenization. A token-level mask ${{\varvec{M}}\in \left\{\text{0,1}\right\}}^{H\times W\times T}$ is used to mix ${x}_{a}$ and ${x}_{b}$ with strong data augmentation, resulting in a new sample ${x}_{mix}$:

4 $$\begin{array}{c}{x}_{{\text{mix}} \, }={\mathcal{A}}_{\text{strong }}\left({x}_{a}\right)\odot M+{\mathcal{A}}_{\text{strong }}\left({x}_{b}\right)\odot \left(1-{\varvec{M}}\right)\end{array}$$

where $1$ is a binary mask consisting of all ones, and the symbol $\odot$ denotes element-wise multiplication.

The training framework for the mixed sample ${x}_{mix}$ is shown in Fig. 4. ${x}_{mix}$ is input into the student model ${\mathcal{F}}_{s}$, resulting in the model prediction ${y}_{mix}={\mathcal{F}}_{s}({x}_{mix})$. The unlabeled video clips ${x}_{a}$ and ${x}_{b}$ are inputted into the teacher model ${\mathcal{F}}_{t}$ using weak data augmentation techniques, which then produce the corresponding pseudo-labels ${\widehat{y}}_{a}$ and ${\widehat{y}}_{b}$ respectively:

5 $$\begin{array}{c}{\widehat{y}}_{a}=\mathit{arg}max\left({\mathcal{F}}_{t}\left({\mathcal{A}}_{\text{weak }}\left({x}_{a}\right)\right)\right)\end{array}$$

6 $$\begin{array}{c}{\widehat{y}}_{b}=arg max\left({\mathcal{F}}_{t}\left({\mathcal{A}}_{\text{weak }}\left({x}_{b}\right)\right)\right)\end{array}$$

It should be observed that if $max\left({\mathcal{F}}_{t}\left({\mathcal{A}}_{\text{weak }}\left(x\right)\right)\right)<\delta$, the pseudo-label $\widehat{y}$ remains as the soft label ${\mathcal{F}}_{t}\left({\mathcal{A}}_{\text{weak }}\left(x\right)\right)$. The pseudo-label ${\widehat{y}}_{mix}$ corresponding to ${x}_{mix}$ is then derived by mixing ${\widehat{y}}_{a}$ and ${\widehat{y}}_{b}$:

7 $$\begin{array}{c}{\widehat{y}}_{\text{mix}}=\lambda \cdot {\widehat{y}}_{a}+\left(1-\lambda \right)\cdot {\widehat{y}}_{b}\end{array}$$

where $\lambda$ serves as the masking ratio for the pseudo-label of the unlabeled sample. Finally, if ${N}_{m}$ represents the number of mixed samples, the hybrid consistency loss of TTMix is obtained to optimize the student model:

8 $$\begin{array}{c}{\mathcal{L}}_{\text{mix }}=\frac{1}{{N}_{m}}\sum^{{N}_{m}}{({\widehat{y}}_{\text{mix}}-{y}_{mix})}^{2}\end{array}$$

### Logit KL divergence standardization preprocessing based on EMA-teacher

#### Unsupervised loss based on logit KL divergence in EMA-teacher

Given the input ${x}_{u}\in {D}_{U}$, the logit prediction vectors of the model of teacher ${\mathcal{F}}_{t}$ and the student ${\mathcal{F}}_{s}$ are ${v}_{uw}={\mathcal{F}}_{t}\left({\mathcal{A}}_{\text{weak }}\left({x}_{u}\right)\right)$ and ${z}_{us}={\mathcal{F}}_{s}\left({\mathcal{A}}_{\text{strong }}\left({x}_{u}\right)\right)$, respectively. Existing methods generally assume that the softmax function converts the prediction vectors into probability vectors ${q(v}_{uw)}$ and ${q(z}_{us})$ using a temperature parameter $\tau$. Specifically, for the k-th entry, the probability is given by:

9 $$\begin{array}{c}q({{\varvec{v}}}_{uw}{)}^{\left(k\right)}=\frac{\mathit{exp}\left({{\varvec{v}}}_{uw}^{\left(k\right)}/\tau \right)}{\sum_{m=1}^{K} \mathit{exp}\left({{\varvec{v}}}_{uw}^{\left(m\right)}/\tau \right)}\end{array}$$

10 $$\begin{array}{c}q({{\varvec{z}}}_{us}{)}^{\left(k\right)}=\frac{\mathit{exp}\left({{\varvec{z}}}_{us}^{\left(k\right)}/\tau \right)}{\sum_{m=1}^{K} \mathit{ex}p\left({{\varvec{z}}}_{us}^{\left(m\right)}/\tau \right)}\end{array}$$

The essence of knowledge distillation is to make $q({{\varvec{z}}}_{us}{)}^{\left(k\right)}$ mimic $q({{\varvec{v}}}_{uw}{)}^{\left(k\right)}$ of all classes or samples, which is achieved by optimizing the loss of KL divergence between the probability distributions:

11 $$\begin{array}{c}{\mathcal{L}}_{KL}\left({q(v}_{uw})||{q(z}_{us})\right)=\sum_{k=1}^{K} q({{\varvec{v}}}_{uw}{)}^{\left(k\right)}log\left(\frac{q({{\varvec{z}}}_{us}{)}^{\left(k\right)}}{q({{\varvec{v}}}_{uw}{)}^{\left(k\right)}}\right)\end{array}$$

In theory, minimizing the KL divergence as described is equivalent to minimizing the cross-entropy loss when optimizing ${\varvec{z}}$ alone:

12 $$\begin{array}{c}{\mathcal{L}}_{CE}({q(v}_{uw}),{q(z}_{us}))=-\sum_{k=1}^{K} q({{\varvec{v}}}_{uw}{)}^{\left(k\right)}log q({{\varvec{z}}}_{us}{)}^{\left(k\right)}\end{array}$$

It should be noted that their gradients are empirically unequal due to the negative entropy term of ${q(v}_{us})$. Considering that in our EMA-Teacher distillation setting, the optimization of the model, i.e., optimizing ${\varvec{z}}$ alone, (2) can be equivalently expressed as the following consistency loss in terms of KL divergence:

13 $$\begin{array}{c}{\mathcal{L}}_{u}=\frac{1}{{N}_{U}}\sum^{{N}_{U}}{\mathbb{I}}\left(max\left(\mathcal{F}\left({x}_{w}\right)\right)>\delta \right){\mathcal{L}}_{\text{KL}}\left({q(v}_{us})||{q(z}_{uw})\right)\end{array}$$

#### Logit-based KL divergence standardization preprocessing in EMA-TEACHER

In (9) and (10), previous methods generally assume that the temperature parameter is shared between the teacher and student models. However, recent research^29^ indicates that there is an inconsistency between different samples and the temperatures of the teacher and student models. They demonstrated that the shared temperature in traditional knowledge distillation algorithms leads to two negative effects: logit shift and variance matching. To address these issues, inspired by their findings, one can enhance the student’s performance by applying a logit standardization preprocessing strategy based on EMA-Teacher KL divergence.

By introducing $\mathcal{Z}$-score preprocessing into (9) and (10), we can adjust the logits before applying the KL divergence. The $\mathcal{Z}$-score standardized logit for a given logit vector from the teacher model can be expressed as:

14 $$\begin{array}{c}q({{\varvec{v}}}_{uw};{{\varvec{v}}}_{uw},\sigma ({{\varvec{v}}}_{uw}){)}^{\left(k\right)}=\frac{\text{exp}(\mathcal{Z}({{\varvec{v}}}_{uw};\tau {)}^{\left(k\right)})}{\sum_{m=1}^{K} \text{exp}(\mathcal{Z}({{\varvec{v}}}_{uw};\tau {)}^{\left(m\right)})}\end{array}$$

15 $$\begin{array}{c}q({{\varvec{z}}}_{us};{{\varvec{z}}}_{us},\sigma ({{\varvec{z}}}_{us}){)}^{\left(k\right)}=\frac{\text{exp}(\mathcal{Z}({{\varvec{z}}}_{us};\tau {)}^{\left(k\right)})}{\sum_{m=1}^{K} \text{exp}(\mathcal{Z}({{\varvec{z}}}_{us};\tau {)}^{\left(m\right)})}\end{array}$$

By introducing $\mathcal{Z}$-score preprocessing into (9) and (10), where a shared temperature parameter $\tau$ is still introduced for both the model of student and teacher, $\mathcal{Z}$ denotes the $\mathcal{Z}$-score function in Algorithm 1. Refer to Algorithm 1 in the Appendix for details. The $\mathcal{Z}$-score standardization process maps both the logits of student and teacher into the same class of Gaussian distribution with a mean of zero and specified standard deviation. This process ensures that the vectors have a zero mean, successfully avoiding the empirical violation of the assumption of a zero mean in previous works^50,54^. Moreover, due to the monotonicity of the $\mathcal{Z}$-score function, the student logits maintain the same scale after transformation as they did originally, preserving and transferring the inherent relationships in the teacher logits to the student model. Additionally, it controls the logit range within $[-\sqrt{K-1}/\tau ,\sqrt{K-1}/\tau ]$, eliminating the possibility of exceedingly large exponentiation values. Thus, Eq. (11) can be rewritten as:

16 $$\begin{array}{c}{\mathcal{L}}_{KD}={\mathcal{L}}_{KL}\left(q({{\varvec{v}}}_{uw};{{\varvec{v}}}_{uw},\sigma ({{\varvec{v}}}_{uw}){)}^{\left(k\right)}||q({{\varvec{z}}}_{us};{{\varvec{z}}}_{us},\sigma ({{\varvec{z}}}_{us}){)}^{\left(k\right)}\right)\end{array}$$

Thus, Eq. (13) can be rewritten as:

17 $$\begin{array}{c}{\mathcal{L}}_{u}=\frac{1}{{N}_{U}}\sum^{{N}_{U}}{\mathbb{I}}\left(max\left(\mathcal{F}\left({x}_{w}\right)\right)>\delta \right){\mathcal{L}}_{\text{KD}}\end{array}$$

Algorithm 2 presents the pseudo-code for applying $\mathcal{Z}$-score standardization preprocessing to the logits within the unsupervised loss, as proposed in this study. Refer to Algorithm 2 in the Appendix for details.

### Adaptive negative learning for mixed samples based on EMA-teacher

In complex scenarios involving human motion videos, the model’s predictions can sometimes be ambiguous. For instance, when the maximum confidence score is only 0.2 but the defined threshold is 0.45, assigning pseudo-labels becomes challenging and ineffective for model optimization. To address this issue, FullMatch^31^ proposed assigning additional labels with less noise to these ambiguous examples to enhance their utility. Their study demonstrated that unlabeled data based on the Top-5 categories is likely to belong to categories other than those previously identified as iterations increase. This observation led to the idea of generating negative pseudo-labels for unlabeled data. Evaluating top-k performance using additional datasets is an ideal approach. By calculating an appropriate k value, it ensures that the error rate for the top k predictions approaches zero. However, using a test set or separating a validation set from labelled data can present practical challenges. As illustrated in the training framework in Fig. 4, we propose an Adaptive Negative Learning (ANL) approach within the EMA-Teacher to approximate the evaluation of Top-k performance.

Specifically, assuming the performance of model can be reflected by the consistency of predictions for various augmented inputs, we first calculate pseudo-labels for mixed samples based on weakly augmented predictions, irrespective of whether the maximum score exceeds the threshold. We then consider these pseudo-labels as the ground truth for the heavily augmented version and calculate the minimum k value such that the accuracy of the top k classifications becomes 100%. This method can be represented as:

18 $$\begin{array}{c}k=arg\underset{\theta \in [2,C]}{min} (Acc({y}_{t\_mix},{\widehat{y}}_{t\_mix},\theta )=100\%)\end{array}$$

Here, ${\widehat{y}}_{t\_mix}$ represents the pseudo-labels of the mixed samples at step t, and ${y}_{t\_mix}$ refers to the prediction vector of the unlabelled data samples after strong data augmentation. $Acc(*)$ and $C$ denote the function for computing the Top-k accuracy and the count of classes, respectively.

Finally, assign these negative pseudo-labels as the categories ranking below the Top-k in the predicted distribution of weakly augmented samples. Thus, for a mixed sample $i$, the selection for a particular category $c$ is given by:

19 $$\begin{array}{c}{s}_{c}^{\left(i\right)}=I\left({y}_{t\_mix}^{i}>\delta \right)+I\left(Rank({y}_{t\_mix}^{i})>k\right)\end{array}$$

Here, $Rank(*)$ is the category ranking function based on descending confidence scores. In the initial stages of the training model, its prediction distributions vary significantly when entering different augmented samples for the same model, which enlarges the value of k. When k = C, ANL does not provide any additional negative pseudo-labels. As the model continues to optimize its cross-entropy loss, its output becomes more invariant to input noise, resulting in a smaller k value and filtering out more usable negative pseudo-labels. The consistency loss is defined as follows:

20 $$\begin{array}{c}{\mathcal{L}}_{anl}=-\frac{1}{{N}_{U}}\sum_{i=1}^{{N}_{U}} \sum_{c=1}^{C} I\left[Rank\left({y}_{{t}_{mix}}^{i}\right)>k\right]log\left(1-{y}_{{t}_{mix}}^{i}\right)\end{array}$$

Here, using ANL does not introduce any additional forward propagation process to evaluate performance, nor does it introduce extra hyperparameters, and it can still assign negative pseudo-labels to ambiguous inputs.

### Training paradigm

The Full-SVFormer training consists of four components: the supervised loss ${\mathcal{L}}_{s}$ in (1), the unsupervised pseudo-label consistency loss ${\mathcal{L}}_{u}$ in (17), hybrid consistency loss ${\mathcal{L}}_{mix}$ of the TTMix in (8), and the adaptive negative learning loss ${\mathcal{L}}_{anl}$ in (20). The training loss for the entire framework ${\mathcal{L}}_{all}$, is given by:

21 $$\begin{array}{c}{\mathcal{L}}_{all}={\mathcal{L}}_{s}+{\gamma }_{1}{\mathcal{L}}_{u}+{\gamma }_{2}{\mathcal{L}}_{mix}+{\mathcal{L}}_{anl}\end{array}$$

where ${\gamma }_{1}$ and ${\gamma }_{2}$ are hyperparameters used to balance the loss terms.

## Experiment

In section A, we first introduce our experimental setup. To ensure comparability with previous work, we conduct experiments under different label rates in section B. Finally, we perform ablation studies and empirical analysis in section C. Throughout the entire experiment, our inference validation uses only the RGB modality on the official dataset.

### Experimental setup

#### Datasets

We conduct experiments on two widely used human action recognition datasets: UCF-101 and HMDB-51, both of which are relatively small in scale. The UCF-101 dataset^61^ is a realistic action video dataset gathered from YouTube. It includes around 13,320 video samples spanning 101 action categories. The videos are grouped into 25 categories, with each group consisting of 4 to 7 action categories. Within the same group, videos may have common features like similar backgrounds or viewpoints. Following the methodology of CMPL^12^, this paper samples 1 or 10 examples per category as the labeled dataset, corresponding to a label rate of 1% or 10%. The HMDB-51 dataset^62^ primarily consists of content sourced from movies, with a smaller portion derived from public databases such as YouTube. As a smaller dataset, it includes 51 action categories comprising approximately 6,766 videos. In line with the partitioning methods of LTG^8^ and Videossl^4^, this paper conducts experiments under three different label rates: 40%, 50%, and 60%.

#### Evaluation metrics

In the experiments, the study primarily uses Top-1 accuracy as the main evaluation metric. Additionally, Top-5 accuracy and model convergence performance are also presented as secondary evaluation metrics, particularly in the ablation experiments.

#### Baseline

The implementation of Full-SVFormer follows the approach detailed in SVFormer^24^, leveraging TimeSformer^15^ with the ViT^23^ baseline architecture as the backbone network. The hyperparameters used in this architecture largely align with the original implementation, employing the TimeSformer’s divided space–time attention mechanism. Because this framework only implements the ViT-base model, Full-SVFormer-S adheres to the implementation method of SVFormer-small. Specifically, it utilizes the DeiT-S^63^ model to develop a Full-SVFormer-small model with a dimension of 384, 6 attention heads, and a depth of 12. This ensures a comparable number of parameters to other convolution-based methods^12,19,38^. For a fair comparison, this study trains the Full-SVFormer for 30 epochs, using it as a supervised baseline.

#### Other details

During the training phase, we adhered to the hyperparameter settings of SVFormer. Specifically, the base learning rate for SVFormer-B was set to 0.005, and for SVFormer-S, it was set to 0.001, with both reduced by a factor of 10 at the 25th and 28th epochs. The confidence threshold $\delta$ was set to 0.3, and the masking ratio $\lambda$ was sampled from a $Beta(\alpha ,\alpha )$ distribution with $\alpha =10$.Due to limited experimental resources, the training was conducted using a single NVIDIA GeForce GTX3090 GPU. The parameters ${\gamma }_{1}$ and ${\gamma }_{2}$ were set to their default values of 1 and 2, respectively, with an introduced temperature parameter of 2, and the extra weight factor for the distillation loss was set to 2. During the testing phase, we followed the inference strategy of SVFormer by uniformly sampling five segments from the entire video and performing three different crops to obtain video frames at a resolution of 224 × 224. This method ensured coverage of most spatial regions within the segments, and the final prediction was derived by averaging the probabilities of softmax of these 5 × 3 predictions.

### Comparison with existing technologies

The main experimental results for UCF-101 are shown in Table 1. At 1% label rate of, Full-SVFormer-S, achieves near-optimal performance with minimal training cost compared to previous methods^41^. Furthermore, when employing the larger model Full-SVFormer-B, it significantly surpasses state-of-the-art methods overall. Specifically, the proposed model, Full-SVFormer-B, improves the Top-1 accuracy by approximately 16.3% compared to the best-performing method^41^ under the 1% label rate, and by about 3.8% compared to the best method^40^ under the 10% label rate. These results highlight a notable performance enhancement achieved by our Full-SVFormer-B model.

**Table 1:** Comparison of state-of-the-art methods on UCF-101.

Method	Epoch	Input	Backbone	w ImgNet	UCF-101(%)	
					1%	10%
FixMatch (NeurIPS 2020)^22^	200	V	SlowFast-R50	√	16.1	55.1
VideoSSL(WACV 2021)^4^	–	V	3D-ResNet-18	√	–	42.0
TCL (CVPR 2021)^7^	400	V	TSM-ResNet-18		–	–
ActorCutMix(CVIU 2021)^39^	600	V	R(2 + 1)D-34	√	–	53.0
MvPL (ICCV 2021)^38^	600	V + F + G	3D-ResNet-50		22.8	80.5
MvPL + ActorSL^40^	800	V + G + Actors	3D-ResNet50		–	82.5
CMPL (CVPR 2022)^12^	200	V	R50 + R50-1/4	√	25.1	79.1
LTG (CVPR 2022)^8^	180/360	V + G	3D-ResNet-18		–	62.4
LTG + ActorSL^40^	800	V + G	3D-ResNet-18		–	65.6
TACL (TCSVT 2022)^64^	200	V	3D-ResNet-50	√	–	55.6
L2A (ECCV 2022)^65^	400	V	3D-ResNet-18	√	–	60.1
ActNetFormer^13^	250	V	3D-ResNet50		27.6	80.6
TimeBalance(CVPR 2023)^41^	150	V	3D-ResNet50		30.1	81.1
Full-SVFormer-S(Ours)	30	V	ViT-S	√	29.4	77.8
Full-SVFormer-B(Ours)	30	V	ViT-B	√	**46.4**	**86.3**

Significant values are in bold.

Notably,"3D-ResNet-18" and "3D-ResNet-50" indicate the backbone networks and their respective depths. During model training, “Input” denotes the data modality, where "V" represents the original video RGB modality, "F" represents the optical flow modality, “Actors” represents annotated entities (i.e. object, animal, or person) that appear in the video, and "G" represents the temporal gradient modality. The performance of different methods in semi-supervised action recognition is evaluated based on Top-1 accuracy.

The main experimental results for the small-scale dataset HMDB-51 are shown in Table 2. Our models, Full-SVFormer-S and Full-SVFormer-B, demonstrate significant improvements in Top-1 accuracy compared to the previous best convolutional-based method^41^. Specifically, for Full-SVFormer-S, the Top-1 accuracy improved by approximately 6.2%, 6.1%, and 5.5% at label rates of 40%, 50%, and 60%, respectively. Notably, Full-SVFormer-B achieved even greater enhancements, with improvements of approximately 13.1%, 13.6%, and 14.9% at label rates of 40%, 50%, and 60%, respectively. It is particularly noteworthy that our models achieved such impressive performance using only the RGB modality as input. Compared to the SITAR^65^ model with Swin Transformer as its backbone network, Full-SVFormer-B reduces model parameters by 37.9% relative to SITAR-L while achieving comparable performance under 50% labeling rate conditions. Furthermore, under labeling rates of 40% and 60%, Full-SVFormer-B demonstrates accuracy improvements of 1.3% and 0.8%, respectively.

**Table 2:** Comparison of state-of-the-art methods on HMDB-51.

Method	Input	Params	Backbone	HMDB-51(%)		
				40%	50%	60%
VideoSSL^4^	V	–	3D-R18	32.7	36.2	37.0
ActorCutMix^39^	V	–	R(2 + 1)D-34	32.9	38.2	38.9
MvPL^38^	V + F + G	–	3D-R18	30.5	33.9	35.8
LTG^8^	V + G	–	3D-R18	46.5	48.4	49.7
TACL^64^	V	–	3D-R18	38.7	40.2	41.7
L2A^65^	V	–	3D-R18	42.1	46.3	47.1
LTG + ActorSL^40^	V + G	–	3D-R18	47.9	50.3	52.4
TimeBalance^41^	V	–	3D-R50	52.6	53.9	54.5
Full-SVFormer-S(Ours)	V	81 M	ViT-S	58.8	60.0	60.9
SITAR-B(ICPR 2025)64^66^	V	87 M	Swin-B	63.4	65.5	68.2
SITAR-L(ICPR 2025)64^66^	V	195 M	Swin-L	64.4	**67.7**	68.6
Full-SVFormer-B(Ours)	V	121 M	ViT-B	**65.7**	67.5	**69.4**

Significant values are in bold.

### Ablation study

Since Full-SVFormer essentially integrates two innovative techniques with SVFormer, it is crucial to perform ablation studies on various components to evaluate their contributions to the model’s performance.

#### Logit standardization preprocessing analysis

**Fig. 5:**
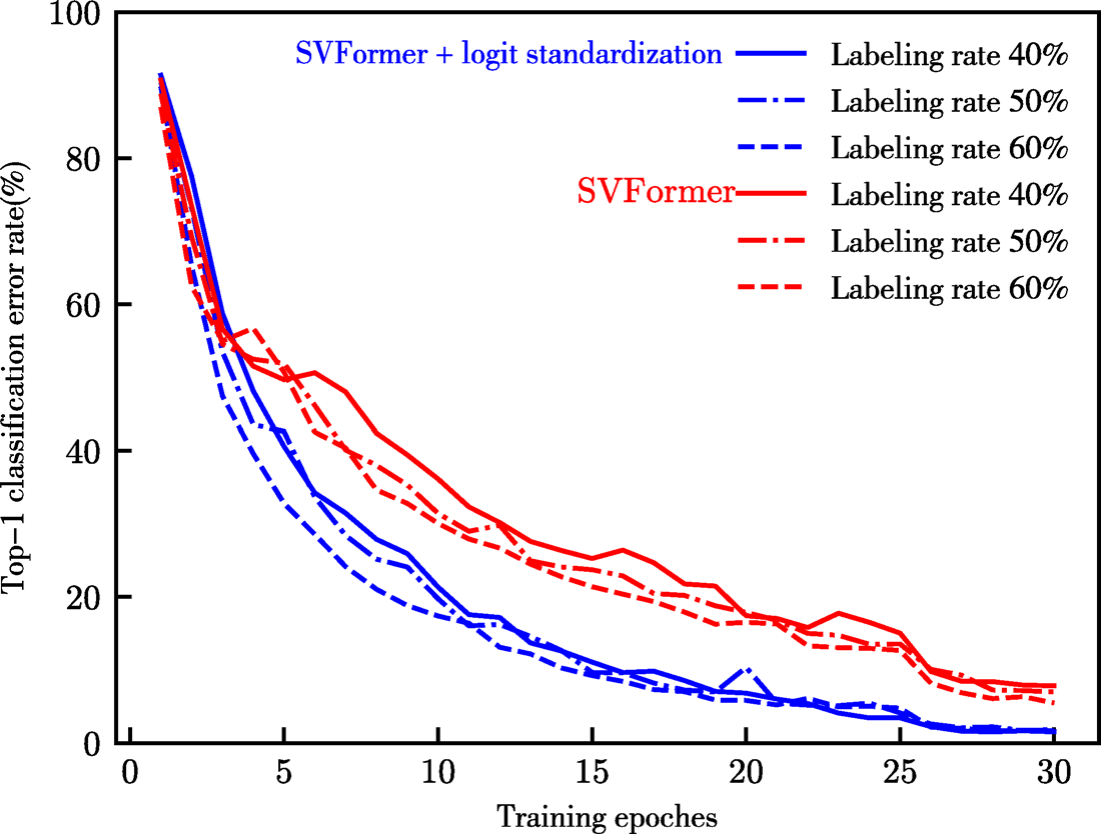
Impact of logit standardization on SVFormer convergence behavior on HMDB-51.

**Fig. 6:**
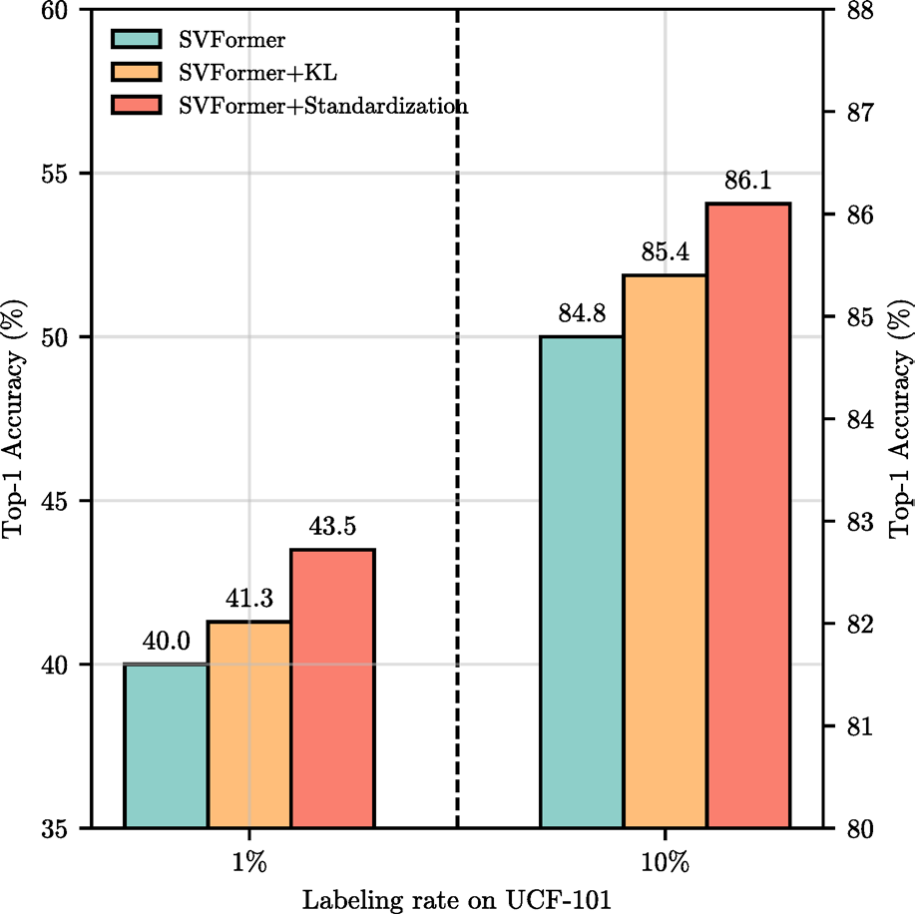
The impact of KL divergence loss and its standardization preprocessing on SVFormer performance.

The comparison results of combining SVFormer with logit standardization preprocessing based on EMA-Teacher’s KL divergence loss are shown in Table 3. The experimental results clearly demonstrate that the introduction of this method significantly enhances the performance of SVFormer. This improvement is attributed to the logit standardization preprocessing, which maps the student’s predicted logits to a bounded range, effectively learning and retaining the inherent relationships of the logit of teacher. It allows the student to focus more on the teacher’s predictions rather than hard labels, alleviating the constraint of forced size matching of the student’s model performance. Notably, there are Top-1 accuracy results on UCF-101 with 1% and 10% labeling rates, and on HMDB-51 with 40%, 50%, and 60% labeling rates. The symbol "Δ" denotes the changes in performance relative to the baseline SVFormer-B, with "+" indicating a performance improvement and "−" indicating a performance decline. As illustrated in Fig. 5, this preprocessing method can accelerate the convergence of model training. Moreover, we observed that the cross-entropy loss, being extremely sensitive to incorrect class labels, might lead to overfitting to noise in the student model. By separately introducing KL divergence loss as the unsupervised consistency loss in Full-SVFormer, which measures the difference between two probability distributions, we can convey the rich information about inter-class correlations and intra-class relationships contained in the teacher model’s soft labels. This acts as a regularization mechanism to prevent the student model from overfitting to noise. Figure 6 demonstrates the improvement in classification performance brought by introducing KL divergence loss as the model’s unsupervised consistency loss.

**Table 3:** Comparison of SVFormer with standardization and ANL.

Method			UCF-101		HMDB-51		
			1%	10%	40%	50%	60%
Standardization ANL			Top-1(%)				
SVFormer-B			40.0	84.8	60.5	63.9	66.3
	**√**		43.5	86.1	65	66.5	67.5
	Δ		** 3.5**	** 1.3**	** 4.5**	** 2.6**	**1.2**
		**√**	39.9	85.2	64.4	65.7	68
	Δ		-0.1	** 0.4**	** 3.9**	** 1.8**	**1.7**
	**√**	**√**	46.4	86.3	65.7	67.5	69.4
	Δ		** 6.4**	** 1.5**	** 5.2**	** 3.6**	**3.1**

Significant values are in bold.

#### Adaptive negative learning (ANL) analysis

**Fig. 7:**
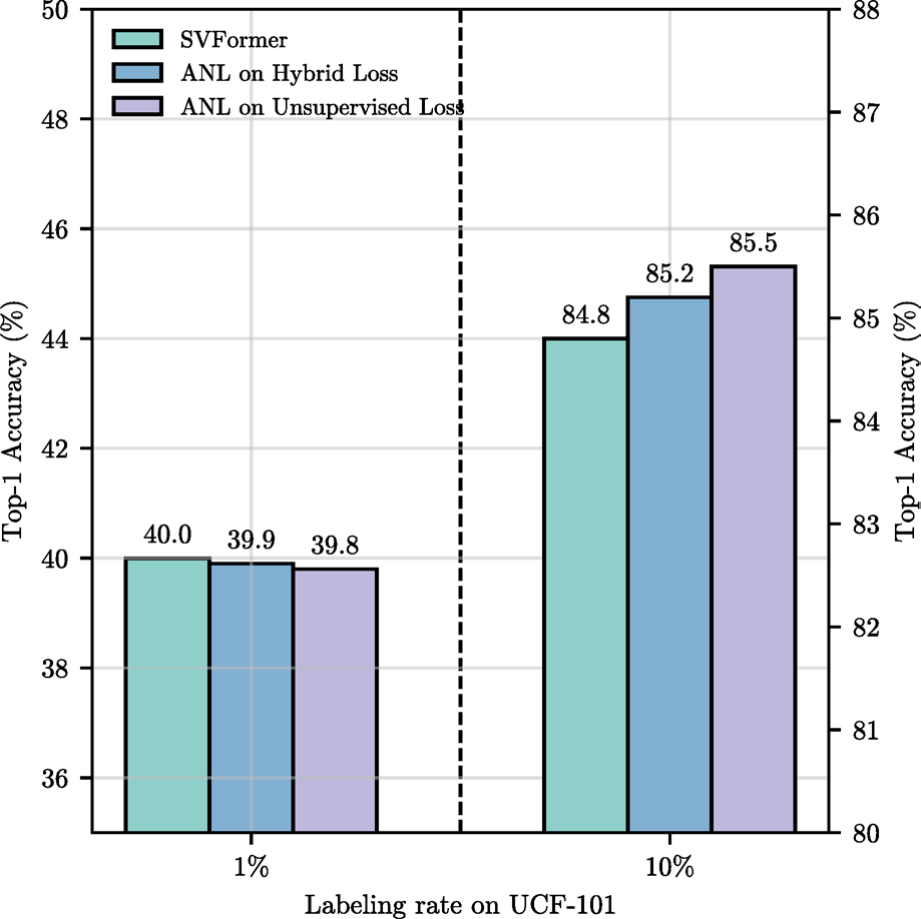
The performance comparison of the Adaptive Negative Learning (ANL) method applied to different modules on the UCF-101 dataset.

The experimental comparison results of combining SVFormer with Adaptive Negative Learning (ANL) are shown in Table 3. From the experimental results, it is clear that the adaptive negative learning method can mitigate the negative impact of incorrect pseudo-labels, effectively improving the performance of model. However, under low labeling rates (such as 1% labeling rate on the UCF-101 dataset), the model’s predictions on unlabeled data are often inaccurate, resulting in pseudo-labels that may contain numerous errors. These erroneous pseudo-labels can adversely affect the model’s training. This paper also applies the Adaptive Negative Learning (ANL) method to two different modules: the unsupervised loss and the mixed loss. Experimental results, as shown in Fig. 7, reveal the following findings: When training with low labeling rates, ANL produces more negative pseudo-labels, causing a slight decrease in performance. However, with high labeling rates, the positive and negative pseudo-labels are used effectively, resulting in improved model performance.

#### Analysis of hyperparameters

In the study, we analyze the effects of the different balanced loss terms. Full-SVFormer adopted different default settings for the balanced loss terms in our training paradigm compared to SVFormer, with ${\gamma }_{1}=1$ and ${\gamma }_{2}=2.$ To provide a more intuitive comparison of the significant improvements our framework offers based on these settings, we conducted an ablation study under this loss balance parameter configuration. As shown in the bench-mark experiment results on HMDB-51 in Table 4, the Full-SVFormer framework demonstrates that these strategies can significantly enhance performance in semi-supervised action recognition overall.

**Table 4:** Comparison between SVFormer-B and Full-SVFormer-B under the training paradigm with ${\gamma }_{1}=1$ and ${\gamma }_{2}=2$.

Method	HMDB-51(%)					
	40%		50%		60%	
	Top-1	Top-5	Top-1	Top-5	Top-1	Top-5
SVFormer-B	62.2	89.9	64.9	91.1	67.2	91.6
Full-SVFormer-B	65.7	91.1	67.5	91.8	69.4	91.3
Δ	** + 3.5**	** + 1.2**	** + 2.6**	** + 0.7**	** + 2.2**	− 0.3

Significant values are in bold.

**Fig. 8:**
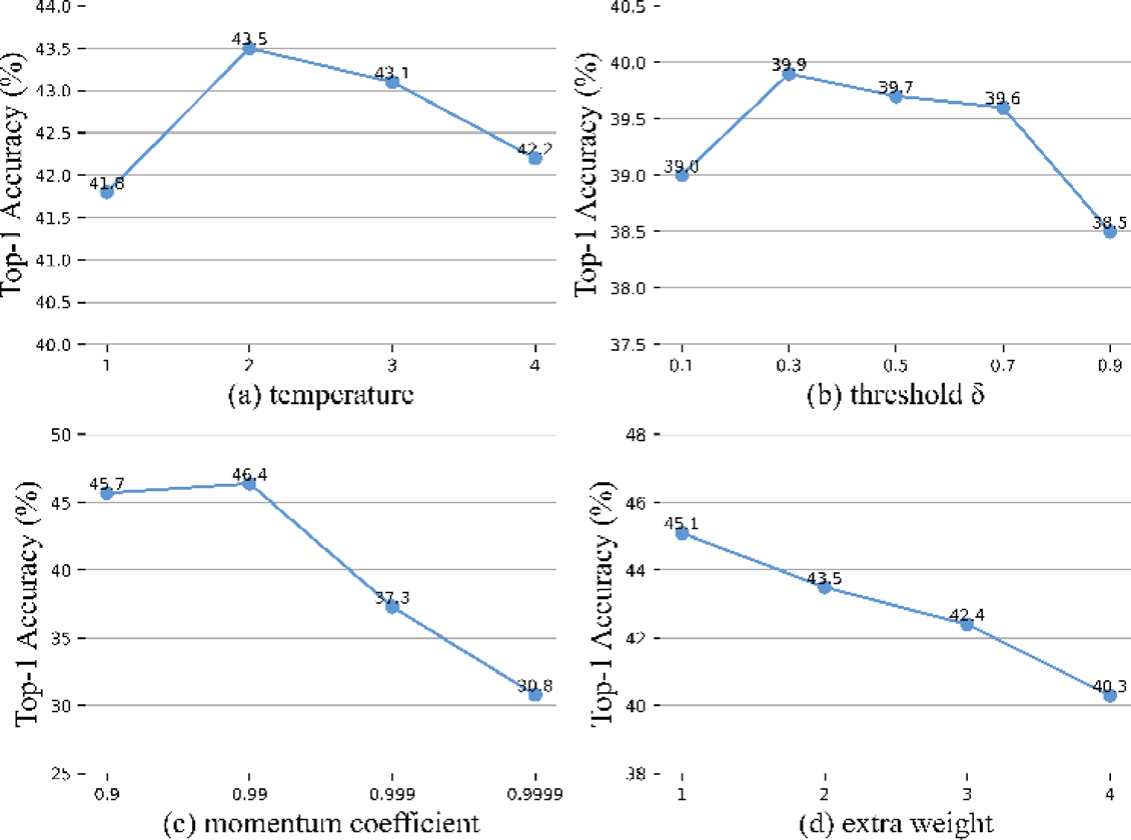
Effects of hyperparameters. The results of varying the distillation temperature, threshold, momentum coefficient, and the extra weight factor of the distillation loss were examined to comprehensively understand the impact of these hyperparameters. The results report the Top-1 accuracy on the UCF-101 dataset with a 1% labeling ratio.

Likewise, we have examined the effects of various other hyperparameters on the model’s performance. The experiments were conducted utilizing the UCF-101 dataset with a 1% labeled data ratio. First, we examined the influence of varying distillation temperatures. From Fig. 8a, it is evident that the best results were obtained when the temperature was set to 2. Then, we assessed the effects with different thresholds for ANL. As shown in Fig. 8b, the optimal result was achieved when its threshold was set to 0.3. After that, we explored the choice of the EMA momentum coefficient and the extra weight of the distillation loss, which is presented in Fig. 8c,d respectively.

The Full-SVFormer model leverages the probability distributions predicted by both the student and the EMA-Teacher models through logit standardization preprocessing applied to the KL divergence-based unsupervised consistency loss. This process smooths high-confidence errors, thereby utilizing the model’s complete output information, which significantly enhances its generalization and feature extraction capabilities.

Additionally, by adaptively constraining negative pseudo-labels within the TTMix mixed sample training framework, the efficiency of unlabeled data utilization and the robustness of model are markedly improved. In Table 3, The experimental results of Top-1 demonstrate that the combination of these methods significantly improves the performance of the semi-supervised action recognition model, Full-SVFormer. This improvement is evident even with limited labeled data, enabling effective action recognition and understanding in videos.

## Conclusion

To address the high labeling costs in the era of socialized video content and the shortcomings of semi-supervised action recognition based on Transformer architecture in this work, we propose a new framework, Full-SVFormer, that uses visual Transformers for semi-supervised action recognition. We introduce a logit standardization preprocessing method for KL divergence within the stable pseudo-label framework, EMA-Teacher, to serve as the consistency loss for semi-supervised action recognition. This innovation eliminates the traditional constraint of forcing the student to match the teacher logits’ magnitude, thereby allowing a focus on the intrinsic relationships of the teacher’s logit. Additionally, we integrate the Adaptive Negative Learning (ANL) method from knowledge distillation, which dynamically evaluates the Top-k performance for all unlabeled samples to assign negative labels. For the performance degradation of the model under low labeling rates when applying the ANL component, we can explore designing a dynamic threshold adjustment mechanism. This mechanism would adjust the confidence threshold based on the current labeling rate to control the generation ratio of negative pseudo-labels. Comparative experiments with traditional CNN extensions in video understanding and several emerging semi-supervised frameworks demonstrate that Full-SVFormer outperforms state-of-the-art techniques in semi-supervised action recognition benchmarks on the HMDB-51 and UCF-101 datasets. Our approach achieves an optimal balance between training costs and classification accuracy for video Transformers, thereby promoting further research into semi-supervised action recognition utilizing Transformer-based architectures. This advancement holds significant potential for future applications in human–computer interaction, sports analysis, medical rehabilitation, and intelligent surveillance.

## Figures and Tables

**Algorithm 1:**
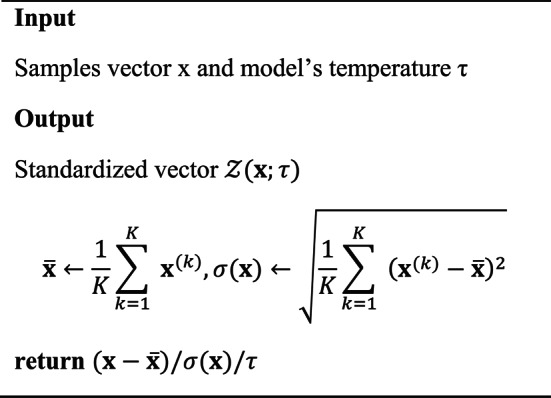
Weighted Z-score function.

**Algorithm 2:**
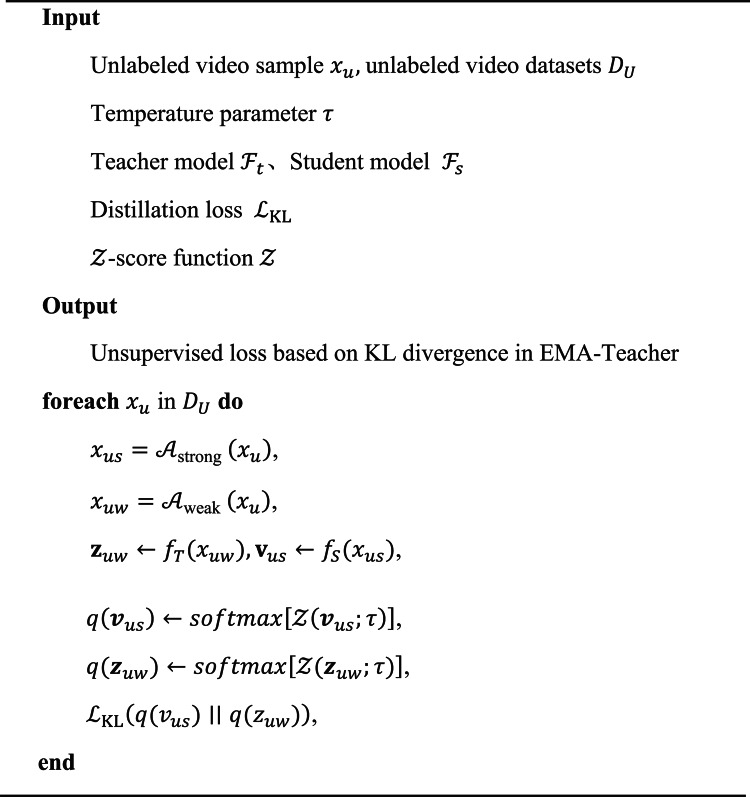
$\mathcal{Z}$-score standardization preprocessing in unsupervised loss based on EMA-Teacher.

## Data Availability

The HMDB-51 dataset can be obtained from https://serre-lab.clps.brown.edu/resource/hmdb-a-large-human-motion-database/, and the UCF-101 dataset can be obtained from https://www.crcv.ucf.edu/data/UCF101.php. All data generated or analyzed during this study are included in this article.

## Appendix

See Algorithms 1 and 2.
